# Roles of Copper-Binding Proteins in Breast Cancer

**DOI:** 10.3390/ijms18040871

**Published:** 2017-04-20

**Authors:** Stéphanie Blockhuys, Pernilla Wittung-Stafshede

**Affiliations:** Department Biology and Biological Engineering, Chalmers University of Technology, 412 96 Gothenburg, Sweden; steblo@chalmers.se

**Keywords:** copper-binding protein, copper transport, lysyl oxidase, SPARC, MEMO1, ATOX1, cancer, breast cancer, metastasis

## Abstract

Copper ions are needed in several steps of cancer progression. However, the underlying mechanisms, and involved copper-binding proteins, are mainly elusive. Since most copper ions in the body (in and outside cells) are protein-bound, it is important to investigate what copper-binding proteins participate and, for these, how they are loaded with copper by copper transport proteins. Mechanistic information for how some copper-binding proteins, such as extracellular lysyl oxidase (LOX), play roles in cancer have been elucidated but there is still much to learn from a biophysical molecular viewpoint. Here we provide a summary of copper-binding proteins and discuss ones reported to have roles in cancer. We specifically focus on how copper-binding proteins such as mediator of cell motility 1 (MEMO1), LOX, LOX-like proteins, and secreted protein acidic and rich in cysteine (SPARC) modulate breast cancer from molecular and clinical aspects. Because of the importance of copper for invasion/migration processes, which are key components of cancer metastasis, further insights into the actions of copper-binding proteins may provide new targets to combat cancer.

## 1. Copper Proteins in Biology

Copper (Cu) is an indispensable metal ion that plays a crucial role in the biochemistry of every living organism. In the body, Cu can exist in oxidized (Cu^2+^) and reduced (Cu^2+^) redox states. The unique electronic structure of Cu makes it useful as cofactor in redox-reactions of enzymes that perform biological functions required for normal growth and development. The recommended daily intake of Cu in healthy adults is 0.9 mg/day. Uptake of Cu in the human body depends on many factors and nutritional components. Cu is absorbed in the small intestine by amino acid transporters mainly for Met, His, and Cys amino acids. Cu is then transported primarily by serum albumin to the liver for subsequent delivery to enzymes and different parts of the body.

The redox activity that allows Cu to contribute functionalities to proteins also provides a risk for toxicity [1]. To minimize toxic effects of free Cu and to regulate the distribution of Cu in time and space, organisms have evolved elaborate protein-based systems for uptake, cellular transport, protein loading, and storing of Cu. During the process of cellular Cu uptake, oxidized Cu is first reduced and then it enters the cell through the copper transporter CTR1 [2]. In the cytoplasm, Cu is shuttled to targets by at least three pathways [3]: in the secretory path, the cytoplasmic Cu chaperone ATOX1 delivers Cu to the Cu-transporting P_1B_-type ATPases ATP7A and ATP7B (Wilson and Menkes disease proteins) in the trans-Golgi network. After transfer to ATP7A/B, fueled by ATP hydrolysis, Cu is channeled to the lumen and loaded onto target Cu-dependent enzymes, e.g., ceruloplasmin (CP) and lysyl oxidase (LOX) [4]. CP is the major Cu transporting protein in blood plasma: it functions as a ferroxidase, oxidizing iron, and it also delivers Cu to other cells [5,6]. In fact, CP contains more than 75% of total plasma Cu, and albumin and transcuprein are known to carry the remaining plasma Cu. LOX is also secreted, and is a Cu-dependent amino oxidase enzyme that crosslinks extracellular matrix (ECM) proteins such as collagen, elastin, and other ECM proteins. In addition to the secretory pathway for intracellular Cu transport, two other pathways for Cu transport in the cytoplasm are known. In one, the Cu chaperone for superoxide dismutase 1 (CCS) delivers Cu to cytoplasmic Cu/Zn superoxide dismutase 1 (SOD1); in the other, Cu is directed to the mitochondria by COX17 and some additional proteins (i.e., SCOs, COX11), for incorporation in cytochrome c oxidase (COX1 and COX2).

We recently established the human Cu proteome (i.e., the collection of all identified human Cu-binding proteins) [7]. We revealed a total of 54 proteins and, of these proteins, 12 are classified as Cu transporters (CTR1 and CTR2 for import, CCS and ATOX1 for cytoplasmic transport, and COX11, COX17, SCO1, and SCO2 that provide Cu to the mitochondria). Also classified as transport proteins are COMMD1 (copper metabolism domain containing 1), an enigmatic protein that may be involved in the regulation of exocytosis of Cu-loaded vesicles [8,9], and cutC copper transporter (CUTC) that, in addition to Cu transport, has been proposed to be an enzyme [10]. About half of the identified Cu-binding proteins are enzymes and these proteins are found in all but Golgi intracellular compartments, extracellularly, and in the plasma membrane. The remaining proteins in the Cu proteome have either non-enzymatic or unknown functions. Notably, none but ATOX1 (see below) of the identified Cu-binding proteins are transcription factors.

## 2. Copper in Cancer

Because Cu is important for the function of many enzymes [1,11,12], it is reasonable that Cu is required for characteristic phenomena involved in cancer progression such as proliferative immortality, angiogenesis, and metastasis [13,14]. In fact, cancer tissue and cancer patients’ serum have been found to contain increased Cu but levels of other metals (e.g., iron, zinc) are often lower than normal [13,15,16]. Cancer is a multifactorial collection of diseases that involves uncontrolled growth of cells, followed by cancer cell invasion, dissemination, and secondary tumor formation at local and distant sites; these processes are often connected with an immune response. For a tumor to grow larger than a few mm, angiogenesis is needed meaning that new blood vessels must form. Cu can induce a pro-angiogenic response [17] by direct binding to angiogenic factors as well as by promoting the expression of such factors [18,19]. Cu can also activate metabolic and proliferative enzymes that enhance the ability of cancerous cells to metastasize, as will be described below [13]. 

Because of the recognized importance of Cu in many of the cancer hallmarks, there have been attempts to develop general Cu-chelating compounds as anticancer therapies [13,20,21]. Reducing systemic Cu levels decreased the activity of COX1 and reduced ATP levels, which leads to lessened oxidative phosphorylation and thereby reduces growth of proliferating cancer cells [22]. Moreover, Cu deprivation was found to inhibit the so-called epithelial-to-mesenchymal transition (EMT) of cells, which is a process in which cells loose cell polarity and cell-cell adhesion and instead gain migratory and invasive properties [23]. However, it is not clear if this type of cell-culture results are transferrable to human cancer patients. Also, as essentially all Cu in the body is protein-bound, drugs targeted to Cu-transport and Cu-dependent proteins important for cancer-promoting processes may constitute more efficient future drug targets. In accordance with this, it was recently shown that small molecules that inhibited cellular Cu transport, by specific targeting of CCS and ATOX1, acted as a selective approach to reducing proliferation of cancer cells but not normal cells [24].

## 3. Copper Proteins Involved in Diverse Aspects of Cancer

For a global view of Cu protein expression changes in various cancers, we recently analyzed the RNA transcript level changes of the Cu proteome (i.e., the identified 54 Cu-binding proteins) in different cancer tissues using information from the Cancer Genome Atlas, or TCGA, database [7]. Many of the proteins in the human Cu proteome exhibit either up- or downregulation in the different cancers [7]. To give some examples, LOX, LOX-like proteins 1 and 2, secreted protein acidic and rich in cysteine (SPARC), and ENOX2 are upregulated in more than 6 out of 18 cancers analyzed. With respect to ATOX1, it is upregulated in breast, colorectal, uterus, and liver tumors [7]. Several studies using sources other than TCGA have also assessed transcript levels in different cancers, but these will not be discussed here. To understand if transcript expression changes (from our analysis and reported by others) are related to cancer-promoting events, molecular studies of defined biological systems are needed. Indeed, some of the enzymes in the Cu proteome have been reported to play mechanistic roles in cancer, as summarized below.

As mentioned earlier, LOX is secreted by cancer cells to create pre-metastatic niches by stimulating collagen cross-linking and fibronectin synthesis [25,26]. Adaptation to hypoxia is a driving force for tumor progression whereby hypoxia stimulates the hypoxia-inducible factor 1α (HIF-1α)-mediated LOX expression [27,28]. Several studies have investigated the mechanistic role of extracellular LOX in cancer cell invasion and metastasis [29,30]. LOX-mediated ECM cross-linking and stiffening induce integrin-mediated focal adhesion formation and PI3K signaling, promoting tumor growth and invasion [31,32]. In addition to extracellular modifications, LOX also appears to regulate cancer progression within cells such as via modulation of actin polymerization that promotes migratory phenotypes [33]. Notably, recent data have indicated LOX and LOX-like proteins as histone-modifying enzymes, which regulate gene expression [34].

MAP2K1 (mitogen-activated protein kinase kinase 1), also called MEK1, is an intra-cellular Cu-dependent kinase involved in the mitogen-activated protein kinase (MAPK) signaling pathway, and thereby is related to cancer growth [35], invasion, and metastasis [36,37]. Another intracellular Cu-dependent protein is mediator of cell motility 1 (MEMO1), a redox enzyme that has been reported to facilitate tumor cell migration and metastasis through several molecular mechanisms. MEMO1 promotes cell migration by stimulating the dynamics of the cell cytoskeleton via activation of cofilin [38] and interaction with the RhoA-mDia1 signaling complex [39] by sustaining reactive oxygen species production upon nitric oxide stimulation in cell protrusions [40], as well as by upregulation of the EMT regulator SNAIL1 through interaction with the insulin-like receptor substrate 1 (IRS1) and activation of the PI3K/Akt signaling pathway [41]. Using a yeast two-hybrid screen, nuclear LOX was found to interact with, among others, MEMO1 [42], suggesting a link between these two Cu-binding proteins. 

SPARC is classified as a secreted Cu-binding glycoprotein with counter-adhesive properties and functions extracellularly to promote invasion. SPARC plays a role in tumor invasion and metastasis via modulation of cell–cell and cell–matrix interactions [43,44]. It was shown that the Cu-binding domain of SPARC mediated cell survival via interactions with integrin β1 and activation of integrin-linked kinase [45]. The role of SPARC seems to depend on the cancer type: it is associated with highly aggressive tumor phenotypes in some cancer [46], but in others it appeared as a tumor suppressor [47]. Taken together, SPARC expression appears to correlate with invasion and progression of gliomas and melanomas but in many epithelial cancers, hyper-methylation of the SPARC promoter reduces the amount of SPARC produced by the tumor cells. A clearer description of the molecular mechanisms of SPARC action is needed to understand its divergent effects on human cancers. Another of the Cu-binding proteins, COMMD1, is interesting as it is often found downregulated in invasive human cancers. It was shown that COMMD1 inhibits both NFκB and HIF-1 mediated gene expression (that both promote tumor growth, survival, and invasion) and, in the case of HIF-1, by direct interaction that disrupted HIF dimerization [48]. Whereas LOX obtains Cu from the secretory pathway, it is not known how SPARC, MEMO1, COMMD1, and MEK1 are loaded with Cu.

Most of the above discussed proteins are classified as Cu-dependent enzymes. However, unprecedented findings have suggested that the Cu chaperone ATOX1 has new activities connected to cancer [35]. ATOX1 was found to act as a Cu-dependent transcription factor [49] that stimulates expression of the proliferation protein cyclin D1 [50] and the extracellular antioxidant protein SOD3 [51,52]. ATOX1 also acts as a Cu-dependent transcription factor for NADPH oxidase promoting inflammatory neovascularization [53] and, ATOX1 may potentially regulate malignant angiogenesis as it is necessary for platelet-derived growth factor-induced Cu-dependent cell migration [54]. Also another cytoplasmic Cu chaperone, CCS, appears to have additional functions in the cell. CCS was shown to enter the nucleus and regulate the HIF-1 transcriptional complex in a Cu-dependent manner. This resulted in expression of the vascular endothelial growth factor (VEGF) which in turn promoted tumor growth [55].

Our research group have also visualized ATOX1 in the nuclei of mammalian cells, but we found no binding to the DNA promotor sequence proposed for ATOX1 upon using in-vitro biophysical experiments [56]. Therefore, we searched for protein partners that may mediate a transcriptional response together with ATOX1. From a large yeast two-hybrid screen, using ATOX1 as bait, we identified several new human protein partners [57] and several of the confident hits are proteins with functions related to signaling and cancer (e.g., CPEB4 (cytoplasmic polyadenylation element binding protein 4) [58], DNMT1 (DNA methyl transferase 1), and PPM1A (protein phosphatase, Mg^2+^/Mn^2+^ dependent 1A) [59,60,61,62]).

## 4. Copper-Binding Proteins in Breast Cancer: A Clinical Perspective

Breast cancer is the most common cancer among women and can be categorized into four molecular subtypes—i.e., luminal A, luminal B, HER2, and triple negative/basal-like—depending on specific biomarkers. Breast cancer tissue and serum have been reported to contain higher Cu levels than control samples and, moreover, the Cu levels were highest in the most advanced breast cancers [63]. Mapping of gene expression signatures in breast cancer cell lines has noted ATP7A as well as LOX as upregulated genes [64]. Also ATP7B was found upregulated in breast cancer but not in normal adjacent tissue [65]. In the latter study, it was suggested that high ATP7B protein levels will increase cell resistance to platinum-based anticancer drugs. Several in vitro studies from our lab have demonstrated that cisplatin, and other platinum compounds, can bind to Cu transport proteins such as ATOX1 and ATP7B [66,67,68]. In accord, it was shown that targeting of cysteine-containing Cu-transporting proteins (e.g., ATP7A/B, ATOX1) with ammonium tetrathiomolybdate enhanced sensitivity of breast cancer cells to cisplatin [69].

Our analysis of the TCGA data for breast cancer [7] revealed upregulation of 26% of the Cu proteome proteins: F5, ATP7B, SLC31A1, SCO2, HEPHL1, CUTA, ATOX1, COX17, TYRP1, MT3, LOX-like proteins 1 and 2, SPARC, and MOXD1. With respect to molecular mechanistic studies of Cu proteins’ roles in breast cancer, there is such information reported for ten proteins (described in detail in Table S1) and below we discuss findings for selected proteins of the ten (MEMO1, SPARC, LOX, and some LOX-like proteins), followed by a separate section about ATOX1.

In approximately 85% of patients with advanced breast cancer, metastasis affects the bone which results in osteolytic lesions and renders the cancer largely untreatable. It was recently demonstrated that LOX secretion was specifically associated with metastasis to the bone in patients with estrogen-receptor negative breast cancer [29]. LOX enzymatic activity promoted TWIST transcription, thereby mediating EMT of cancer cells [70]. Moreover, in breast cancer cells, LOX was found intracellularly, both in the nucleus and cytoplasm, and hydrogen peroxide generated as a side-product upon LOX activity appeared to facilitate cell adhesion and migration through activation of the FAK/Src signaling pathway [71].

LOXL2, LOXL3, and LOXL4 all belong to the LOX-like (LOXL) family of proteins. Like LOX, LOXL2 was shown to promote invasive/metastatic phenotypes in breast cancer cells which was explained by altered LOXL2 protein processing and localization [72]. It was found that LOXL2 and LOXL3 interacted with, and stabilized, SNAIL1, which induced EMT and promoted invasion through repression of, among others, E-cadherin expression [73]. In addition to SNAIL1 effects, LOXL2 influences expression of SPARC and the extracellular proteins TIMP1 and MMP9 [74,75]; in fact, LOXL2 was noted as prognostic factor in breast cancer [74]. LOXL2 activity in basal-like carcinoma cells were found to affect tight junction and cell polarity complexes by a mechanism which involves downregulation of involved genes and, LOXL2 is required for cell invasion, tumor growth, and lung metastasis of basal-like breast carcinoma cells [76].

As already mentioned in the previous section, MEMO1 facilitates tumor cell migration, metastasis, and pathways related to EMT, and these results were obtained using breast cancer cells [39,40,41]. Because many breast cancer types are hormone-dependent it is important to probe cross-interactions between growth factor- and steroid hormone-mediated signaling pathways in order to find suitable drug targets. In this respect, MEMO1 was reported to act at the intersection between growth factor (heregulin and IGF1) and estrogen signaling in breast cancer cells. Specifically, MEMO1 was found to control estrogen receptor α (ERα) sub-cellular localization, phosphorylation, and function downstream of ErbB2/ER or IGFIR/ER thereby activating MAPK and PI3K signaling pathways that promoted breast cancer cell migration and/or proliferation [77,78].

SPARC stimulates breast cancer growth and metastasis in in-vivo models [79]. Statistical analysis of tissue microarray data showed that upregulation of SPARC occurs in basal-like breast tumors [80]. Moreover, SPARC levels were found to be inversely correlated with the content of the estrogen receptor. This result suggests that SPARC levels are associated with more aggressive breast cancer tumors [81,82].

## 5. ATOX1 in Breast Cancer

Mapping of gene expression signatures in breast cancer cell lines has noted upregulation of ATOX1 via proteomics [83]. Using immunohistochemistry, we investigated ATOX1 in 67 breast cancer sections in tissue microarrays (TMAs). In agreement with the TCGA data (mentioned above, [7]), we found ATOX1 levels to be increased in cancer as compared to normal breast tissue. Scoring of the 67 breast cancer samples revealed that the highest ATOX1 intensities were found in samples of all cancer molecular subtypes but the HER2 subtype [7]. When we turned to cell line studies, we stumbled on a putative functional role for ATOX1 in breast cancer cells. Using an aggressive breast cancer cell line, we made the discovery that ATOX1 accumulates at lamellipodia borders of migrating breast cancer cells and ATOX1 silencing resulted in migration defects as evidenced from reduced wound closure. Thus, ATOX1 may have an unknown role in breast cancer cell migration [84], that parallels the reported role for ATOX1 in endothelial cell wound healing [85].

Interestingly, CPEB4 has been reported to play a promoting role in breast cancer by modulating mRNA transcript translation in the cytoplasm [58]. As mentioned above, our yeast two-hybrid screen identified an interaction between CPEB4 and ATOX1 [57] which calls for further investigation of putative synergistic cancer-promoting effects between these proteins.

## 6. Summary and Outlook

**Figure 1:**
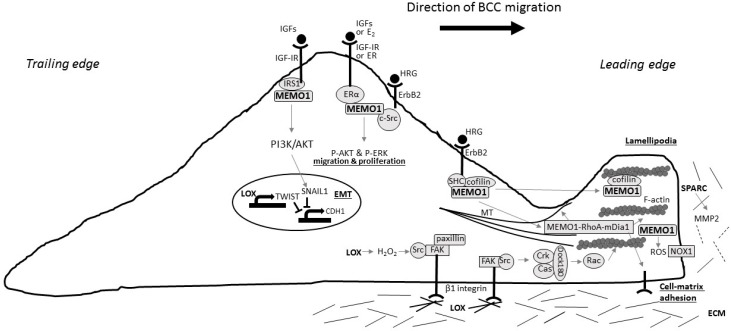
Model of a migrating breast cancer cell (BCC) with reported molecular signaling pathways for LOX, SPARC, and MEMO1. (IGF, insulin growth factor; IGF-IR, insulin growth factor 1 receptor; E2, estrogen; ER, estrogen receptor; HRG, heregulin; IRS1, insulin receptor substrate 1; ERα, estrogen receptor α; PI3K, phosphoinositide 3-kinase; EMT, epithelial-mesenchymal transition; MT, microtubuli; ROS, reactive oxygen species; NOX1, NADPH oxidase 1; ECM, extracellular matrix; MMP2, matrix metalloproteinase 2; FAK, focal adhesion kinase); SHC, Src homology 2 domain containing. The arrows indicate the direction of the molecular signaling pathways.

Clearly, Cu-dependent processes are of importance for breast (and other) cancer development. Thus it becomes important to elucidate the molecular mechanisms and pathways for how involved Cu-dependent proteins are loaded with Cu—i.e., how the flow of Cu via Cu transport proteins are directed to Cu-dependent proteins in breast cancer cells. Based on the available data for Cu-binding proteins in breast cancer, speculations can be made that, of course, should be tested experimentally in the future in controlled cell lines [86]. In Figure 1, we have compiled the known paths involving the Cu-binding proteins LOX, MEMO1, and SPARC in breast cancer, using a migrating cancer cell as the model.

One interesting possibility is that ATOX1 delivers Cu to MEMO1 at the lamellipodia edges such that MEMO1 becomes activated and, in turn, can activate cofilin (direct interaction between MEMO1 and cofilin has been reported [38]) resulting in actin dynamics modulation and thereby promotion of cell migration. We further speculate that this intra-cellular scenario may be coupled to integrin-mediated ECM-induced signaling, e.g., from SPARC, which can stimulate small GTPases that can play roles in cofilin/actin function. We imagine that a wealth of new molecular, mechanistic knowledge in the coming years will allow for the development of new breast cancer drugs directed towards selected Cu-binding proteins.

## Supplementary Materials

Supplementary materials can be found at www.mdpi.com/1422-0067/18/4/871/s1.
