# Supplementary material for: Roles of Copper-Binding Proteins in Breast Cancer
Source: Int J Mol Sci. 2017 Apr 20;18(4):871. doi: 10.3390/ijms18040871 (PMC5412452; doi:10.3390/ijms18040871)
Supplement: Supplementary file 1 [file ijms-18-00871-s001.pdf]

Supplemental Material

Roles of copper-binding proteins in breast cancer

By Stephanie Blockhuys and Pernilla Wittung-Stafshede

Table S1

(legend, table, references)

Table S1

Summary of molecular mechanistic studies of Cu-binding proteins in breast cancer, including the following columns 'CBP' (i.e., abbreviated names of the Cu-binding proteins, CBP), 'study model' (*i.e.*, in vitro and/or in vivo; cell line studies and/or mice models), 'treatment' (*i.e.*, genetic and/or chemical), 'process (+/-)' (*i.e.*, the related breast cancer cell process, which is positively or negatively related with the CBP's expression, 'mechanism' (*i.e.*, details about the functional and/or molecular mechanism), and 'Ref.' (*i.e.*, literature references). (ATOX1, antioxidant 1 copper chaperone; LOX, lysyl oxidase; LOXL2, LOX-like 2; LOXL4, LOX-like 4; SPARC, secreted protein acidic and rich in cysteine; MEMO1, mediator of cell motility 1; MT3, metallothionein 3; MAP2K1, Mitogen-Activated *Protein* Kinase Kinase 1; PARK7, Parkinson disease protein 7; LTF, lactoferrin; βAPN, beta-aminopropionitrile; BCS, bathocuprione disulphonate; TIMP1, tissue inhibitor of metalloproteinase-1; MMP9/3, matrix metalloproteinase-9/3; s.c., subcutaneous; i.v., intravenous; MDSC, myeloid-derived suppressor cells; IRS1, insulin receptor substrate 1; TM, tetrathiomolybdate; AO, anti-sense oligonucleotides; KD, knockdown; MICS, membrane invasion culture system; MSC: mesenchymal stem cells).

CBP	Study model		Treatment	Process	Mechanism	Ref.
ATOX1	In vitro	MDA-MB-231 in wound-healing assay	ATOX1 KD (siRNA)	migration (+)	ATOX1 accumulates in lamellipodia borders.	1
LOX	In vitro	MDA-MB-231, Hs578T (highly invasive/ metastatic) and MCF7, T47D (low invasive/non-metastatic) in MICS assay	LOX KD (AO), LOX activity inhibition (βAPN), LOX expression (*mLOX* gene)	invasion (+)	Upregulated LOX expression in metastatic compared with non-metastatic cell lines.	2
	In vitro	MDA-MB-231, Hs578T (highly invasive/ metastatic) and MCF7 (low invasive/non-metastatic) in cell-matrix adhesion, Cell Motility HitKit, and modified MICS assays.	LOX activity inhibition (βAPN), LOX expression (LOX-His32/50 DNA).	adhesion, motility, migration (+)	LOX facilitates migration through regulation of cell-matrix adhesion formation. Intracellular LOX -> H_2O_2 production -> FAK/Src pathway activation.	3
	In vitro	MDA-MB-231, Hs578T (highly invasive/ metastatic) and MCF7 (low invasive/non-metastatic)	LOX activity inhibition (βAPN), LOX expression (LOX-His32/50 DNA).	-	LOX regulates actin polymerization and thus lamellipodia formation. LOX (-> FAK/Src) -> $p130^{Cas}$/Crk/DOCK180 pathway -> Rac activity	4
	In vitro	MCF-7, MDA-MB-231, AU-565, BT-483 (cancerous), MCF-10A, HBL-100 (normal) in wound-healing and Transwell invasion (Matrigel) assays.	Inhibition of LOX activity (βAPN), and LOX synthesis (RNAi, Magnolol)	Migration, invasion (+)	Upregulated LOX expression in metastatic compared with non-metastatic cancerous and normal cell lines. LOX -> FAK/paxillin signaling complex	5
	In vitro	MDA-MB-231 in wound-healing and Transwell invasion (Matrigel coated and uncoated) assays.	Inhibition of LOX protein expression (shRNA) and LOX mRNA (AO), inhibition of LOX activity (βAPN, BCS or antibody).	Migration, invasion, (+)	Hypoxia -> secreted LOX -> FAK activity and cell-matrix adhesion.	6
	In vivo	MDA-MB-231 injected *s.c.* (orthotopic model) and in tail veins (lung metastatic tumor model) of nude mice.		Metastasis (+)		
	In vitro	MDA-MB-231, MCF7, MCF7/Ras, T47D in Transwell and collagen-coated microchannels migration assays.	Inhibition of LOX activity (βAPN) or expression (shRNA)	Migration (+)	Extracellular hyaluronan causes nuclear translocation of CD44 in the cancer cells, thus triggering LOX transcription and, in turn, LOX stimulates Twist transcription which mediates EMT. (MSCs-triggered EMT and metastasis)	7
	In vivo	MDA-MB-231 and MCF7/Ras *s.c.* injected in nude mice.		Growth, metastasis (+)		
	In vivo	MDA-MB-231 injected intracardial in NIH-III female mice.	Inhibition of LOX activity (βAPN)	Metastasis (+)	-	8
LOXL2	In vitro	MCF-7 (cancerous), MCF-10A (normal)	Overexpression of LOXL2 (cDNA)	Migration (+)	LOXL2 induces increase in migration ability of MCF-7 but not MCF-10A, related with altered LOXL2 localization and processing in these two cell lines.	9
	In vitro	MCF7, MDA-MB-231, SkBR3, BT474, BT549 (tumorigenic); HBL100 (non- tumorigenic).	Inhibition of LOXL2 expression (shLOXL2)	Migration, invasion (+)	LOXL2 expressed in basal-like cell lines (MDA-MB-231, BT549, HBL100). LOXL2 KD induces a MET phenotype and suppresses metastasis.	10

	In vivo	MDA-MB-231 injected in mammary fat pad (orthotopic model) or in tail vein (lung metastatic tumor model) of BALB/c nude mice.		Growth, metastasis (+)	LOXL2 negatively modulates the expression and organization of tight junctions and cell polarity complexes, by transcriptional repression of claudin1 and Lgl2 genes, resp., independent of Snail1.	
	In vitro	MDA-MB-231, HBL100, BT549, HS578T (basal-like); MCF7, MDA-MB-361, BT474, T47D (Er+/PgR+ luminal), SK-BR3 (Her2-positive) in wound-healing and Transwel invasion (Matrigel) assays	LOXL2 KD (siRNA).	Migration, invasion (+)	LOXL2 expression specific for basal-like BCCs. LOXL2 promotes EMT and invasiveness of basal-like BBCs. LOXL2 contributes positively to FAK/SRC activation and influences Snail, Snai2, and SPARC expression.	11
	In vitro	MDA-MB-231 and 4T1 in Transwell invasion (Matrigel) assay.	Inhibition of LOXL2 genetically (shRNA), chemically (D-penicillamine) or antibody-mediated.	Invasion (+)	LOXL2 regulates the expression and activity of the extracellular proteins TIMP1 and MMP9.	12
	In vivo	MDA-MB-231 and 4T1 injected in mammary fat pad of immunocompetent syngeneic BALB/c mice and immunocompromised nude mice, respectively.		Metastasis (+)		
LOXL4	In vitro	MDA-MB-231 in Transwell migration (uncoated) and invasion (Matrigel) assays	LOXL4 KD (siRNA)	migration, invasion (+)	Weak LOXL4 leads to ECM remodeling, *i.e.*, altered synthesis, deposition, structure of collagen and increased bundle thickness.	13
	In vivo	MDA-MB-231 injected in mammary fat pad (orthotopic model) or in tail vein (lung metastatic model) of BALB/c nude mice		growth, metastasis (+)		
SPARC	In vitro	MDA-MB-231, BT549 (invasive); MCF-7 (non-invasive)	rSPARC and SPARC peptides	-	SPARC plays role in collagen-induced activation of MMP2 (and proteolysis) at cell surface of the invasive cancer cell lines (might be due in part to diminution of TIMP2 protein).	14
	In vitro	MCF-7 (non-invasive) in Transwell motility (uncoated) and invasion (Matrigel) assays	Stable SPARC overexpression (MCF7/SPARC); Stable c-Jun overexpression (c-Jun/MCF7); Inhibition of SPARC expression in c-Jun/MCF7 (AO).	Motility, invasion (+)	SPARC plays an important role in stimulating motility and invasive behavior of c-Jun/MCF7 cells, but overexpression of SPARC in MCF7 is not sufficient to promote cell migration and invasion.	15
	In vitro	MDA-MB-231 in wound-healing assay	SPARC expression induced by doxycycline treatment of SPARC transfected MDA-MB-231 BAG cells (using Tet-On inducible system)	Proliferation (-)	SPARC slows cell cycle progression to S phase.	16

	In vitro	MDA-MB-231 in Transwell invasion (Matrigel) assay	SPARC expression (cDNA)	Invasion (-)	High expression of SPARC in MDA-MB-231 inhibits tumor cell-platelet interactions, which combined with the reduced invasion, contributes to the decreased metastasis of these cells.	17
	In vivo	MDA-MB-231 injected intracardially in female athymic nude mice		Metastasis (-)		
	In vitro	MDA-MB-231 in wound-healing or Transwell migration assay.	SPARC protein immunopurified from MDA-MB-468, HBME-1, or hFOB cell-conditioned media.	Migration (+)	SPARC induces undirected breast cancer cell motility, through its anti-adhesive properties.	18
	In vivo	Murine 4T1 and LM3 breast malignant cells implanted in syngeneic BALB/c mice.	SPARC KD (shRNA)	Growth, metastasis (+)	SPARC induces primary tumor growth by enhancing cell cycle and by promoting a COX-2-mediated expansion of MDSC. SPARC facilitates metastasis by a COX-2-independent enhancement of cell disengagement from the primary tumor and adherence to the lungs that fostered metastasis implantation.	19
MEMO1	In vitro	MDA-MB-231, T47D, SKBr3 in Transwell migration (Col-I coated) assay.	MEMO1 or Shc KD (siRNA); stable expression of myc-MEMO1 in SKBr3 (pcDNA); ErbB2 inhibitor PKI166	Migration (+)	MEMO1 facilitates MT outgrowth. Upon HRG-mediated activation of ErbB2, MEMO1 interacts with phospho-Tyr 1227 of ErbB2 receptor through Shc adaptor protein.	20
	In vitro	T47D, NYPD, SKBr3, MDA-MB-435 in Transwell migration (Col-I coated) assay.	MEMO1, PLCγ1, or cofilin KD (siRNA)	Migration (+)	MEMO1 increases the actin-polymerizing and actin-severing activity of cofilin. Upon HRG-mediated activation of ErbB2, MEMO1 interacts with cofilin and influences PLCγ and cofilin activities.	21
	In vitro	T47D, SKBr3 in random motility assay.	MEMO1 or mDia1 KD(siRNA)	Migration (+)	Memo –RhoA – mDia1 signaling coordinates the organization of lamellipodial actin network, adhesion site formation, and MT outgrowth within the cell leading edge. Upon HRG-mediated activation of ErbB2, MEMO1 contributes to localize the small G protein RhoA and its effector mDia1 to the plasma membrane.	22
	In vitro	T47D in Transwell migration (Col-I coated) and proliferation assays.	MEMO1 KD (shRNA); expression of myc-MEMO1 (pcDNA)	Migration, proliferation (+)	Upon HRG (ErbB2) and E2 (oestrogen receptor) stimulation, MEMO1 interacts with Src and ERα, which results in increased Y418-Src, Y537-ERα and extra-nuclear retention of ERα. (MAPK and PI3K/Akt signaling pathway activation)	23

	In vitro	MCF7, ZR75-1, T47D (ER-positive) and SKBR3 (ER-negative) in anchorage-dependent and –independent growth assays	MEMO1, IGF1R or ERBB2 KD (siRNA), E_2 treatment, E_2 antagonist (Tamoxifen, ICI182,780)	Growth (+)	MEMO1 interacts with IGF-IR and ErbB2, and mediates extra-nuclear function of ER, including activation of MAPK and PKB/AKT, and integration of function with nuclear ER.	24
	In vivo	MCF7 or ZR75-1 injected in mammary fat pad of female nude mice.				
	In vitro	MDA-MB-231, MCF10A (IGF1R-positive); SKBr3, BT474 (IGF1R-negative/HER2-positive) in 3D colony formation (on Matrigel and soft-agar), and Transwell migration and invasion (Matrigel) assays.	MEMO1, IRS1, or Snail KD (shRNA); MCF10A-MEMO1 (pcDNA); IGF-I treatment	Proliferation, migration, invasion (+)	MEMO1 interacts with IRS1 which leads to PI3K/AKT signaling pathway activation and further Snail1 upregulation (triggers EMT program).	25
	In vitro	MDA-MB-231, T47D, SKBr3 in wound healing and Transwell invasion (Matrigel) assays	MEMO1 KD or reconstituted expression (Myc-MEMO) (shRNA)	Migration, invasion (+)	MEMO1 sustains ROS production in response to NOX1 activation in the lamellae.	26
	In vivo	MDA-MB-231 injected in mammary fat pad (orthotopic model) or tail vein (lung metastatic tumor model) of nonobese diabetic/ severe combined immunodeficient mice		Metastasis (+)		
MT3	In vitro	MCF-7, MDA-MB-231, (MT3 negative), MDA-MB-231/BO2 (MT3 overexpressing), SK-BR-3, and BT-474 in Transwell invasion (Matrigel) assay	MT3 overexpression (pcDNA); MT3 and MMP KD (siRNA)	Invasion (+)	MT3 overexpression increases BCC invasion via upregulation of MMP3 activity.	27
	In vivo	Cells injected s.c. in female athymic Crl:NU-Foxn1$_{nu}$ mice (nude mice)		-	-	
MEK1	In vitro	MDA-MB-231 in Transwell migration (bFGF) and FN-adherence assays	MEK1/2 KD (siRNA), MEK inhibitor (PD184352), AKT inhibitor (AKTi), block MEK1-AKTs interaction (MEK1 peptides), EGF stimulation	Migration and adhesion (+)	MEK1/2 - AKT complex phosphorylates the migration-related transcription factor FoxO1	28
	In vivo	MDA-MB-231 injected *s.c.* in fat pad of CD-1 nude mice.	*i.v.* injection with MEK peptide or MEK inhibitor (PD184352)	Metastasis (+)		

PARK7	In vitro	MCF-7, T47D, MDA-MB-231, MDA-MB-435 in Transwell invasion (Matrigel) assay	PARK7 KD (siRNA); PARK7 or KLF overexpression (pcDNA); Ras inhibition (LY294002 or PD98059)	Invasion (+)	PARK7 represses KLF17 expression and thereby negatively regulates the KLF17/ID-1 pathway. PARK7 acts as EMT-positive regulator (downregulating E-cadherin and increasing Snail). PARK7 regulates cell invasion in Ras-dependent manner.	29
LTF	In vitro	MDA-MB-231, MCF-7 in thymidine uptake assay	LTF protein (supplement in culture medium)	Proliferation (-)	LTF treatment induces growth arrest at G1 to S transition of cell cycle by modulating expression and activity of key G1 regulatory proteins.	30
	In vitro	MDA-MB-231 in Transwell migration (uncoated) assay	LTF expression (pcDNA)	Migration (-)	-	31

References

1. Blockhuys, S.; Wittung-Stafshede, P. Copper chaperone Atox1 plays role in breast cancer cell migration. Biochem. Biophys. Res. Commun. **2017**, 483, 301-304. doi: 10.1016/j.bbrc.2016.12.148.
2. Kirschmann, D.A.; Seftor, E.A.; Fong, S.F.; Nieva, D.R.; Sullivan, C.M.; Edwards, E.M.; Sommer, P.; Csiszar, K.; Hendrix, M.J. A molecular role for lysyl oxidase in breast cancer invasion. Cancer Res. **2002**, 62, 4478-4483. doi:10.1158/0008-5472.CAN-10-2868
3. Payne, S.L.; Fogelgren, B.; Hess, A.R.; Seftor, E.A.; Wiley, E.L.; Fong, S.F..; Csiszar, K.; Hendrix, M. J.; Kirschmann, D.A. Lysyl oxidase regulates breast cancer cell migration and adhesion through a hydrogen peroxide–mediated mechanism. Cancer Res. **2005**, 65, 11429-11436. doi:10.1158/0008-5472.CAN-05-1274
4. Payne, S.L.; Hendrix, M.J.; Kirschmann, D.A. Lysyl oxidase regulates actin filament formation through the $p130^{Cas}$/Crk/DOCK180 signaling complex. J Cell Biochem. **2006**, 98, 827-837. doi:10.1002/jcb.20792
5. Chen, L.C; Tu, S.H.; Huang, C.S.; Chen, C.S.; Ho, C.T.; Lin, H.W.; Lee, C.H.; Chang, H.W.; Chang, C.H.; Wu, C.H.; et al. Human breast cancer cell metastasis is attenuated by lysyl oxidase inhibitors through down-regulation of focal adhesion kinase and the paxillin-signaling pathway. Breast Cancer Res. Treat. **2012**, 134, 989-1004. doi:10.1007/s10549-012-1986-8
6. Erler, J.T.; Bennewith, K.L.; Nicolau, M.; Dornhöfer, N.; Kong, C.; Le, Q.T. ; Chi, J. T.; Jeffrey, S.S.; Giaccia, A.J. Lysyl oxidase is essential for hypoxia-induced metastasis. Nature **2006**, 440, 1222-1226. doi:10.1038/nature04695
7. El-Haibi, C.P.; Bell, G.W.; Zhang, J.; Collmann, A.Y.; Wood, D.; Scherber, C.M.; Csizmadia, E.; Mariani, O.; Zhu, C.; Campagne, A.; et al. Critical role for lysyl oxidase in mesenchymal stem cell-driven breast cancer malignancy. Proc. Natl. Acad. Sci. U S A. **2012**, 109, 17460-17465. doi:10.1073/pnas.1206653109
8. Bondareva, A.; Downey, C.M.; Ayres, F.; Liu, W.; Boyd, S.K.; Hallgrimsson, B.; Jirik, F.R. The lysyl oxidase inhibitor, β- aminopropionitrile, diminishes the metastatic

colonization potential of circulating breast cancer cells. PloS One **2009**, 4, e5620. doi: 10.1371/journal.pone.0005620
9. Hollosi, P.; Yakushiji, J.K.; Fong, K.S.; Csiszar, K.; Fong, S. Lysyl oxidase-like 2 promotes migration in noninvasive breast cancer cells but not in normal breast epithelial cells. Int. J. Cancer. **2009**, 125, 318-327. doi: 10.1002/ijc.24308
10. Moreno-Bueno, G.; Salvador, F.; Martín, A.; Floristán, A.; Cuevas, E.P.; Santos, V.; Montes, A.; Morales, S.; Castilla, M.A.; Rojo-Sebastián, A.; et al. Lysyl oxidase-like 2 (LOXL2), a new regulator of cell polarity required for metastatic dissemination of basal-like breast carcinomas. EMBO Mol.Med. **2011**, 3, 528-544. doi: 10.1002/emmm.201100156.
11. Ahn, S.G.; Dong, S.M.; Oshima, A.; Kim, W.H.; Lee, H.M.; Lee, S.A.; Kwon, S.H.; Lee, J.H.; Lee, J.M.; Jeong, J.; et al. LOXL2 expression is associated with invasiveness and negatively influences survival in breast cancer patients. Breast Cancer Res. Treat. **2013**, 141, 89-99. doi: 10.1007/s10549-013-2662-3.
12. Barker, H.E.; Chang, J.; Cox, T.R.; Lang, G.; Bird, D.; Nicolau, M.; Evans, H.R.; Gartland, A.; Erler, J.T. LOXL2-mediated matrix remodeling in metastasis and mammary gland involution. Cancer Res. **2011**, 71, 1561-1572. doi:10.1158/0008-5472.CAN-10-2868
13. Choi, S.K.; Kim, H.S.; Jin, T.; Moon, W.K. LOXL4 knockdown enhances tumor growth and lung metastasis through collagen-dependent extracellular matrix changes in triple-negative breast cancer. Oncotarget **2017**. doi: 10.18632/oncotarget.14450.
14. Gilles, C.; Bassuk, J.A.; Pulyaeva, H.; Sage, E. H.; Foidart, J.; Thompson, E.W. SPARC/osteonectin induces matrix metalloproteinase 2 activation in human breast cancer cell lines. Cancer Res. **1998**, 58, 5529-5536. PMID:9850090
15. Briggs, J.; Chamboredon, S.; Castellazzi, M.; Kerry, J. A.; Bos, T. J. Transcriptional upregulation of SPARC, in response to c-Jun overexpression, contributes to increased motility and invasion of MCF7 breast cancer cells. Oncogene **2002**, 21, 7077-7091. doi: 10.1038/sj.onc.1205857.
16. Dhanesuan, N.; Sharp, J.A.; Blick, T.; Price, J.T.; Thompson, E.W. Doxycycline-inducible expression of SPARC/osteonectin/BM40 in MDA-MB-231 human breast cancer cells results in growth inhibition. Breast Cancer Res. Treat. **2002**, 75, 73-85. PMID:12500936
17. Koblinski, J.E.; Kaplan-Singer, B.R.; VanOsdol, S.J.; Wu, M.; Engbring, J.A.; Wang, S.; Goldsmith, C.M.; Piper, J.T.; Vostal, J.G.; Harms, J.F.; et al. Endogenous osteonectin/SPARC/BM-40 expression inhibits MDAMB231 breast cancer cell metastasis. Cancer Res. **2005**, 65, 7370-7377. doi: 10.1158/0008-5472.CAN-05-0807.
18. Campo McKnight, D.A.; Sosnoski, D.M.; Koblinski, J.E.; Gay, C.V. Roles of osteonectin in the migration of breast cancer cells into bone. J. Cell. Biochem. **2006**, 97, 288-302. doi:10.1002/jcb.20644.
19. Guttlein, L.N.; Benedetti, L.G.; Fresno, C.; Spallanzani, R.G.; Mansilla, S.F.; Rotondaro, C.; Raffo Iraolagoitia, X.L.; Salvatierra, E.; Bravo, A.I.; Fernández, E.A.; et al. Predictive outcomes for HER2-enriched cancer using growth and metastasis signatures driven by SPARC. Mol. Cancer Res. **2017**, 15, 304-316. doi: 10.1158/1541-7786.MCR-16-0243-T.
20. Marone, R.; Hess, D.; Dankort, D.; Muller, W.J.; Hynes, N.E.; Badache, A. Memo mediates ErbB2-driven cell motility. Nat. Cell. Biol. **2004**, 6, 515-522. doi:10.1038/ncb1134
21. Meira, M.; Masson, R.; Stagljar, I.; Lienhard, S.; Maurer, F.; Boulay, A.; Hynes, N.E. Memo is a cofilin-interacting protein that influences PLCgamma1 and cofilin activities,

and is essential for maintaining directionality during ErbB2-induced tumor-cell migration. J. Cell. Sci. **2009**, 122, 787-797. doi: 10.1242/jcs.032094.
22. Zaoui, K.; Honoré, S.; Isnardon, D.; Braguer, D.; Badache, A. Memo-RhoA-mDia1 signaling controls microtubules, the actin network, and adhesion site formation in migrating cells. J. Cell. Biol. **2008**, 183, 401-408. doi: 10.1083/jcb.200805107.
23. Frei, A.; MacDonald, G.; Lund, I.; Gustafsson, J.Å.; Hynes, N.E.; Nalvarte, I. Memo interacts with c-Src to control estrogen receptor alpha sub-cellular localization. Oncotarget. **2016**, **7**, 56170-56182. doi: 10.18632/oncotarget.10856.
24. Jiang, K.; Yang, Z.; Cheng, L.; Wang, S.; Ning, K.; Zhou, L.; Lin, J.; Zhong, H.; Wang, L.; Li, Y.; et al. Mediator of ERBB2-driven cell motility (MEMO) promotes extranuclear estrogen receptor signaling involving the growth factor receptors IGF1R and ERBB2. J. Biol. Chem. **2013**, 288. 24590-24599. doi: 10.1074/jbc.M113.467837.
25. Sorokin, A.V.; Chen, J. MEMO1, a new IRS1-interacting protein, induces epithelial-mesenchymal transition in mammary epithelial cells. Oncogene **2013**, 32, 3130-3138. doi: 10.1038/onc.2012.327.
26. MacDonald, G.; Nalvarte, I.; Smirnova, T.; Vecchi, M.; Aceto, N.; Dolemeyer, A.; Frei, A.; Lienhard, S.; Wyckoff, J.; Hess, D.; et al. Memo is a copper-dependent redox protein with an essential role in migration and metastasis. Sci. Signal. **2014**, **7**, ra56. doi: 10.1126/scisignal.2004870.
27. Kmiecik, A.M.; Pula, B.; Suchanski, J.; Olbromski, M.; Gomulkiewicz, A.; Owczarek, T.; Kruczak, A.; Ambicka, A.; Rys, J.; Ugorski, M.; et al. Metallothionein-3 increases triple-negative breast cancer cell invasiveness via induction of metalloproteinase expression. PloS One **2015**, 10, e0124865. doi: 10.1371/journal.pone.0124865.
28. Procaccia, S.; Ordan, M.; Cohen, I.; Bendetz-Nezer, S.; Seger, R. Direct binding of MEK1 and MEK2 to AKT induces Foxo1 phosphorylation, cellular migration and metastasis. Sci. Rep. **2017**, **7**, 43078. doi: 10.1038/srep43078.
29. Ismail, I.A.; Kang, H.S.; Lee, H.J.; Kim, J.K.; Hong, S.H. DJ-1 upregulates breast cancer cell invasion by repressing KLF17 expression. Brit. J. Cancer. **2014**, 110, 1298-1306. doi: 10.1038/bjc.2014.40.
30. Damiens, E; El Yazidi, I.; Mazurier, J.; Duthille, I.; Spik, G.; Boilly-Marer, Y. Lactoferrin inhibits G1 cyclin-dependent kinases during growth arrest of human breast carcinoma cells. J. Cell. Biochem. **1999**, 74, 486-498. PMID:10412049.
31. Vecchi, M.; Confalonieri, S.; Nuciforo, P.; Viganó, M.A.; Capra, M.; Bianchi, M.; Nicosia, D.; Bianchi, F.; Galimberti, V.; Viale, G.; et al. Breast cancer metastases are molecularly distinct from their primary tumors. Oncogene **2008**, 27, 2148-2158. doi: 10.1038/sj.onc.1210858.
